# Supplementary material for: Causal Relationship between Aging and Anorexia Nervosa: A White-Matter-Microstructure-Mediated Mendelian Randomization Analysis
Source: Biomedicines. 2024 Aug 16;12(8):1874. doi: 10.3390/biomedicines12081874 (PMC11351342; doi:10.3390/biomedicines12081874)
Supplement: Supplementary file 1 [file biomedicines-12-01874-s001.zip › Supplementary Figure/Figure S4/Singe SNP plot of MR analysis for TL to ubm-b-1880.pdf]

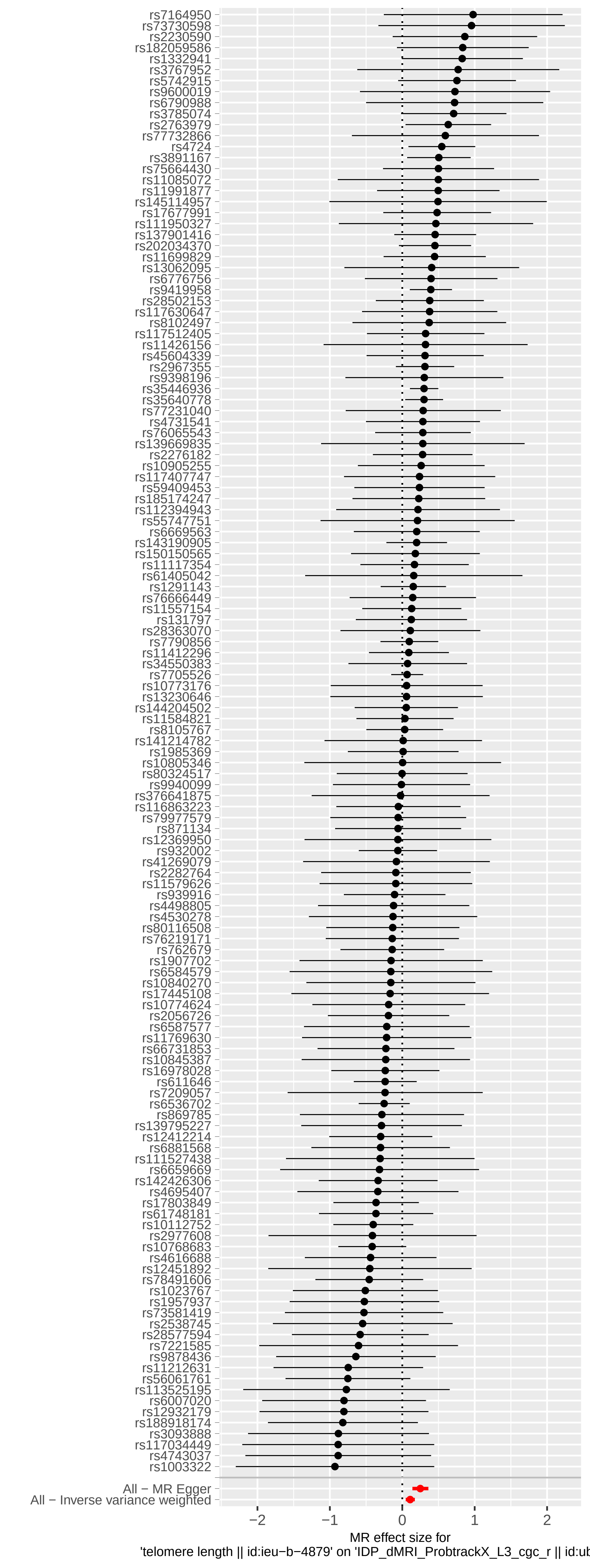
rs7164950
rs73730598
rs2230590
rs182059586
rs1332941
rs3767952
rs5742915
rs9600019
rs6790988
rs3785074
rs2763979
rs77732866
rs4724
rs3891167
rs75664430
rs11085072
rs11991877
rs145114957
rs17677991
rs111950327
rs137901416
rs202034370
rs11699829
rs13062095
rs6776756
rs9419958
rs28502153
rs117630647
rs8102497
rs117512405
rs11426156
rs45604339
rs2967355
rs9398196
rs35446936
rs35640778
rs77231040
rs4731541
rs76065543
rs139669835
rs2276182
rs10905255
rs117407747
rs59409453
rs185174247
rs112394943
rs55747751
rs6669563
rs143190905
rs150150565
rs11117354
rs61405042
rs1291143
rs76666449
rs11557154
rs131797
rs28363070
rs7790856
rs11412296
rs34550383
rs7705526
rs10773176
rs13230646
rs144204502
rs11584821
rs8105767
rs141214782
rs1985369
rs10805346
rs80324517
rs9940099
rs376641875
rs116863223
rs79977579
rs871134
rs12369950
rs932002
rs41269079
rs2282764
rs11579626
rs939916
rs4498805
rs4530278
rs80116508
rs76219171
rs762679
rs1907702
rs6584579
rs10840270
rs17445108
rs10774624
rs2056726
rs6587577
rs11769630
rs66731853
rs10845387
rs16978028
rs611646
rs7209057
rs6536702
rs869785
rs139795227
rs12412214
rs6881568
rs111527438
rs6659669
rs142426306
rs4695407
rs17803849
rs61748181
rs10112752
rs2977608
rs10768683
rs4616688
rs12451892
rs78491606
rs1023767
rs1957937
rs73581419
rs2538745
rs28577594
rs7221585
rs9878436
rs11212631
rs56061761
rs113525195
rs6007020
rs12932179
rs188918174
rs3093888
rs117034449
rs4743037
rs1003322
All − MR Egger
All − Inverse variance weighted
−2
−1
0
1
2
MR effect size for
'telomere length || id:ieu−b−4879' on 'IDP_dMRI_ProbtrackX_L3_cgc_r || id:ub
